# Children With Intracranial Arachnoid Cysts: Classification and Treatment

**DOI:** 10.1097/MD.0000000000001749

**Published:** 2015-11-06

**Authors:** Zhen Tan, Yongxin Li, Fengjun Zhu, Dongdong Zang, Cailei Zhao, Cong Li, Dan Tong, Heye Zhang, Qian Chen

**Affiliations:** From the Department of Pediatric Neurosurgery, ShenZhen Children Hospital, ShenZhen (ZT, FZ, DZ, CL, DT, QC); Institute of Anatomy, Southern Medical University, GuangZhou (YL); Department of Pediatric Radiology, ShenZhen Children Hospital (CZ); and Shenzhen Institutes of Advanced Technology, Chinese Academy of Sciences, Shenzhen, China (HZ).

## Abstract

We performed a dynamic study of arachnoid cysts (ACs) using magnetic resonance cisternography (MRC) and proposed a classification of ACs.

Twenty-three suitable patients in our hospital entered into this study according to our inclusion criteria. MRC images were collected in all the subjects at 1 and 24 hours after the administration of intrathecal gadolinium-diethylenetriamine penta-acetic acid (Gd-DTPA). We allocate the enrolled patients into 2 groups, MRC group and surgery group. The MRC results were considered before treatment in 1 group (MRC group, 13 patients), whereas another group was surgically treated without considering the MRC results (surgery group, 10 patients). We calculated the enhanced area of cyst using modified MacDonald Criteria from the images and measured the surrounding subarachnoid area as the reference.

We found that it was practically useful to quantify 3 types of ACs, complete communicating, incomplete communicating, and noncommunicating, according to MRC results in this study. All the subjects in both groups are closely observed before the treatment and the follow-up using the MRI examination. In the surgery group, 5 patients were found that the area of cysts shrank in the follow-up stage. However, there was no significant difference in the percentage shrinkage area between the 2 groups.

We concluded that MRC with Gd-DTPA as a contrast agent is of significant clinical value for the diagnosis and treatment of children with intracranial ACs. This classification based on dynamic MRC is useful for making surgical recommendations.

## INTRODUCTION

Arachnoid cysts (ACs) are the benign malformations of the arachnoid, which can gradually destroy the primitive arachnoid membrane. The continuous development of ACs always leads to intraarachnoid fluid collection inside the brain.^1,2^ Surgical treatment for ACs is a controversial issue. In the most of previous studies, symptomatic ACs associated with hydrocephalus, seizure, increased intracranial pressure, and focal neurologic deficits were recommended to take surgically treatment.^3,4^ However, in daily clinical practice, a number of symptomatic patients with ACs were found to have unrelieved symptoms after the removal of ACs. One study reported that only about 60% of surgical cases have significant clinical improvement.^5,6^ More recent studies have shown that it is necessary to accurately assess the characteristics of intraarachnoid fluid, which is the analysis of the communication between the cyst and surrounding subarachnoid space, would help clinicians make surgical decisions for different patients.^7^ Therefore, it is necessary to prove that dynamic characteristics of the fluid flow within ACs may be more meaningful than clinical symptoms when a surgical decision is made.

Magnetic resonance cisternography (MRC), which uses low-osmolality paramagnetic gadolinium as an intrathecal contrast agent in the setting of enhanced magnetic resonance myelography/cisternography, is a safer, less invasive, and more radioactive technique compared with other cisternographic (radionuclide or computed tomography cisternography [CTC]) tests. It provides all the advantages of magnetic resonance imaging (MRI),^8^ and it can yield both morphologic and dynamic information.^9,10^ MRC has shown that cisternography and ventriculography gadolinium-diethylenetriamine penta-acetic acid (Gd-DTPA)-enhanced MRI is a feasible and useful technique for the evaluation of obstructions and communications of the subarachnoid space, spontaneous or traumatic/postsurgical craniospinal cerebrospinal fluid (CSF) leaks, or postsurgical adhesions/arachnoiditis in the pediatric population.^11^

In the current study, MRC with Gd-DTPA was used to assess the dynamic characteristics of symptomatic ACs and to classify these ACs into 3 groups. On the basis of the classification, different patients were treated with different methods. By comparing the percentage shrinkage of different groups during follow-up, we sought to prove that the use of MRC with Gd-DTPA as a contrast agent is of significant clinical value for the diagnosis and treatment of children with intracranial ACs, and that this classification based on dynamic MRC is useful for surgical decision-making.

## MATERIALS AND METHODS

### Patients

Between December 2013 and February 2015, 23 patients were diagnosed with ACs by radiology imaging in our hospital, and all of them were symptomatic. After obtaining signed informed consent forms, which were approved by the Shenzhen Children Hospital Ethical Review Board, all 23 patients were enrolled in this prospective study.

We used sealed envelopes to perform randomization. Twenty-three symptomatic ACs were distributed into 2 groups at random, the MRC group and the surgical group. MRC results were considered before treating 13 patients (MRC group), and a surgical plan was formulated according to the MRC classification. Another 10 patients (surgical group) who composed the control group were surgically treated without any consideration of the MRC results. The grouping was described, and the clinical characteristics, such as age, sex, size, cyst location, and symptoms, were compared between the 2 groups (Table 1). The cyst volume was measured pre- and postoperation with the methods described below.

**TABLE 1:**
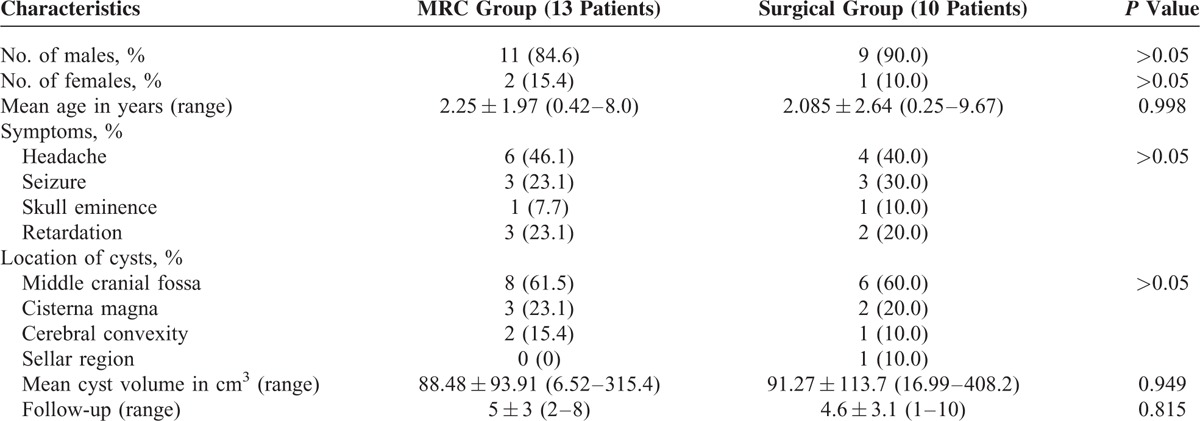
Comparison of Baseline Demographic Characteristics Between the 2 Study Groups

### Cisternography/Ventriculography

All children under 5 years of age took or received an enema of 10% chloral hydrate (0.5 mL/kg) for sedation before the cisternography or ventriculography. Patients with an unclosed anterior fontanelle underwent lateral ventricle puncture through the anterior fontanelle. Patients with a closed anterior fontanelle underwent lumbar puncture.

### Lumbar Puncture

All the subjects were required to keep in the lateral decubitus position. One lumbar puncture using a No. 6 needle was implemented at the L3–L4 level to collect 5 mL of cerebrospinal fluid. Under sterile conditions, the cerebrospinal fluid was mixed with 0.5 mL of Gd-DTPA (Magnevist, Bayer Faerie, Germany). Then, the mixed liquid was injected into the lumbar cistern through the puncture needle at a rate of 1 mL/min. The patients were advised to lie supine with their feet higher than their head for 10 to 20 minutes. After cisternography or ventriculography (except for MRI examination), the patients received continuous electrocardiogram (ECG) monitoring for 24 hours. The children's vital signs and other incident symptoms and signs were also observed.

### Lateral Ventricular Puncture Through the Anterior Fontanelle

The patients lay supine. After skin preparation and disinfection, the lateral ventricle was punctured at the right angle of the anterior fontanelle angle with a needle (No. 6), perpendicular to the imaginary line between the bilateral external auditory canals. When the clinician had a breakthrough sensation, the needle core was withdrawn. The outflow of cerebrospinal fluid indicated successful puncture. Five milliliters of cerebrospinal fluid was extracted during this process. Under sterile conditions, the cerebrospinal fluid was mixed with 0.5 mL of Gd-DTPA (Magnevist, Bayer Faerie, Germany). Then, the mixed liquid was injected into the lateral ventricle through the puncture needle at the rate of 1 mL/min. The patients were advised to lie supine with their feet higher than their head for 10 to 20 minutes. After cisternography or ventriculography (except for MRI examination), the patients received continuous ECG monitoring for 24 hours. The children's vital signs and other incident signs and symptoms were observed.

### MRI Examination

Before the cisternography or ventriculography, the patients had routine MRI scans including T1-weighted images (TR/TE/NEX = 380–460/8–17/3) and T2-weighted images (2000/30–90/1) on 3 orthogonal planes with a 1.5T MRI machine (Signa, HiSpeed, GE, Milwaukee, WI). One hour after successful puncture and injection, MRI was carried out again. The scan parameters were the same as those before contrast infusion. Twenty-four hours after the cisternography or ventriculography, MRI was carried out again. All MRI data were observed and diagnosed by 2 neuroimaging physicians.

### Cyst Volume Measurement

**FIGURE 1:**
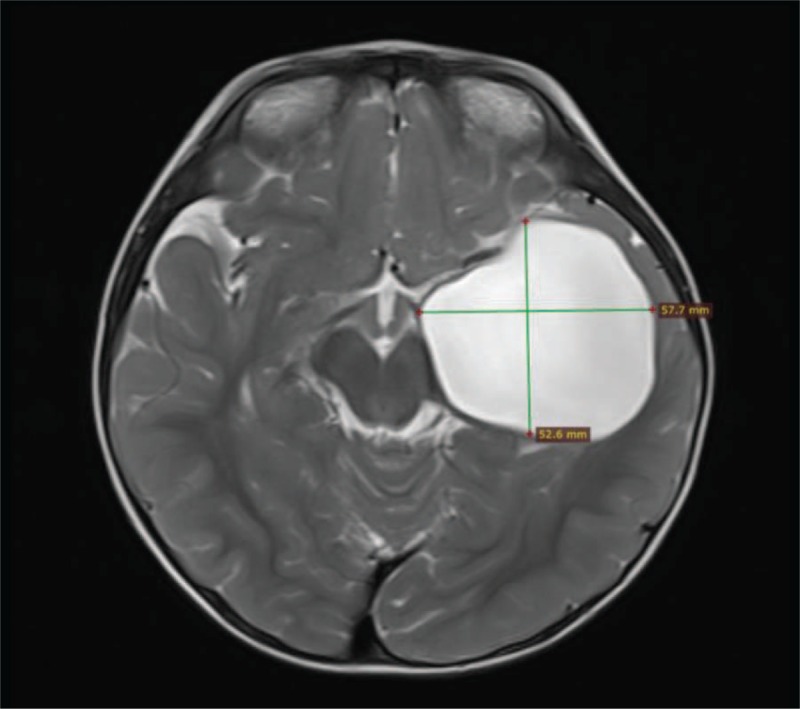
Modified MacDonald method to measure cyst volumes.

The modified MacDonald Criteria are applied for volume measurement in this study.^12^ According to the modified MacDonald Criteria, we measure the volume through the following steps: the number of image slices in which the cyst is visible and the maximum cross-sectional and orthogonal diameters of the cyst. We selected the slice with the largest area of cyst, and measured 2 longest orthogonal diameters of the cyst area (Fig. 1). These 2 diameters, the number of slices in which the cyst was present, and the slice thickness plus the gap were used to compute the volume by using the following formula:

Cyst volume = πd1d2(s × t)/6

where d1 and d2 are the orthogonal diameters, s the number of cyst slices, and t is the slice thickness.

### Angiographic Diagnostic Criteria

Neuroimaging physicians compared and analyzed the T1-weighted MRI data from 1 and 24 hour after the cisternography or ventriculography. By comparing the signals of the cyst and cistern at 1 and 24 hour, the cysts were classified.

## TREATMENT

Surgical treatment was performed in the patients with incomplete communicating and noncommunicating cysts in the MRI group and all patients in the surgical group. Endoscopic fenestration or microscopic fenestration was performed under general anesthesia. Microscopic fenestration was performed through a minimal skin incision and a small minicraniotomy. After opening the dura, the outer cyst wall was partially incised to penetrate the inner cyst wall and enter the basal cisterns. Several windows between the cyst and the cisterns were made to enable a smooth flow of cystic fluid or CSF. The dura was closed by primary repair or duraplasty with an artificial dura mater. The bone flap was repositioned with an absorbable strut. A drain catheter was not inserted in the majority of cases. Endoscopic fenestration was conducted after 1 burr hole was made. A rigid neuroendoscope was used for the fenestration procedure. After the insertion of the endoscope into the cyst cavity, several fenestrations between the cyst and the basal cistern (or ventricle) were made. At the end of the procedure, the dura was closed. The burr hole site was typically not repaired.

### Follow-Up

During the follow-up stage, all the patients were required to take MRI examination. Then, the volume of cyst in each patient was measured and recorded accordingly.

## RESULTS

### Patients and Comparability Between the 2 Groups

Age, sex, size and location of cyst, symptoms, and follow-up time were shown in Table 1.

### AC Classification Based on MRC

**FIGURE 2:**
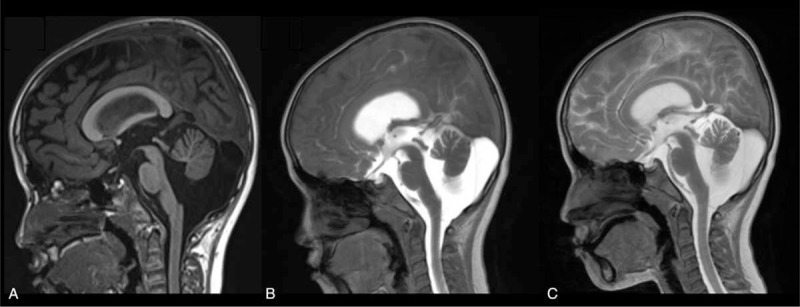
AC in the cistern magna of an 11-year-old male. An arachnoid cyst located in the cistern magna was unexpectedly found during CT after a head injury. The cyst did not cause any symptoms. One hour after cisternography, the cyst was enhanced in the sagittal T1 sequence (B). Sagittal T1 at 24 hours showed the signal inside the cyst was equal to the surrounding cistern, indicating a complete communicating AC (C). AC = arachnoid cyst, CT = computed tomography.

**FIGURE 3:**
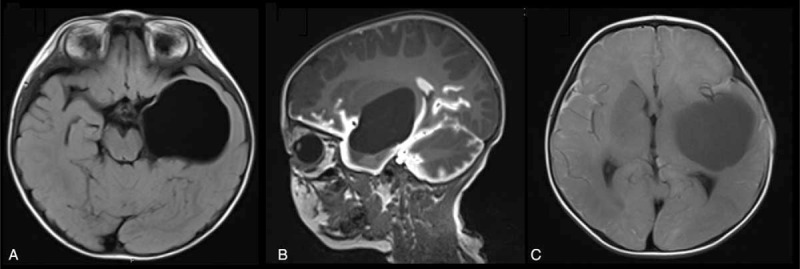
Arachnoid cyst (AC) in the left middle fossa of a 1.5-year-old male. The patient presented with a left temporal bone eminence without other symptoms. One hour after contrast injection, sagittal T1 demonstrated enhancement of the surrounding cistern instead of the cyst (B). Axial T1 at 24 hours showed a higher signal inside the cyst than that in the lateral ventricle, suggestive of incomplete communicating AC (C).

**FIGURE 4:**
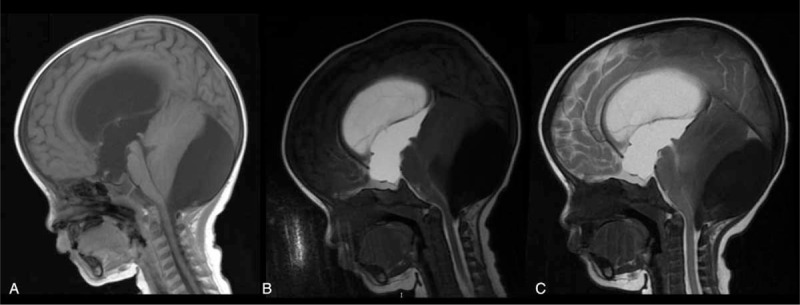
Arachnoid cyst (AC) in the cistern magna of a male 1 year and 3 months of age. The patient presented with retardation of motor development. Physical examination revealed that the muscle force of the lower limbs was grade IV. One hour after contrast injection, sagittal T1 showed imaging of the 4th ventricle and the spinal subarachnoid space and no immediate imaging of the cyst (B). Sagittal T1 at 24 hours showed imaging of the 4th ventricle and the subarachnoid space surrounding the cyst. As the signal of the cyst was lower than the cistern and ventricle, he was diagnosed with noncommunicating AC (C).

One 5-month-old boy received lateral ventricle puncture through the anterior fontanelle, whereas the remaining 12 patients received lumbar puncture. After MRC, the characteristics of cyst imaging at different time points were summarized (Figs. 2–4 and Table 2). Based on MRC diagnostic criteria, ACs were classified into 3 types: complete communicating cyst, incomplete communicating cyst, and noncommunicating cyst (Table 3).

**TABLE 2:**
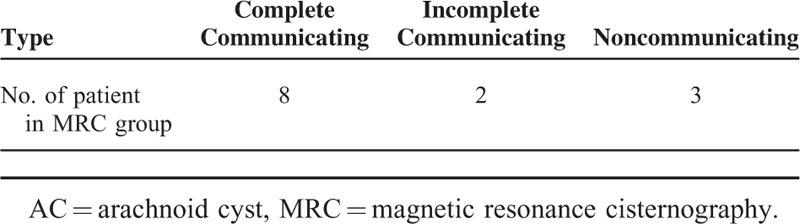
ACs Classification Based on MRC

**TABLE 3:**
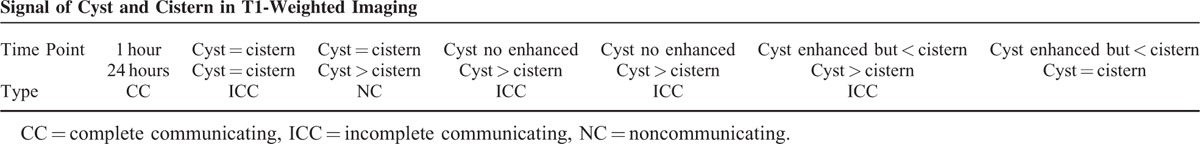
Angiographic Diagnostic Criteria Based on Signal in Cyst and Cistern

During the cisternography, no patients developed severe complications. The only side effect was transient mild headache in an 8-year-old patient. The symptom was relieved after bed rest and oral administration of analgesics. After the cisternography, no abnormalities in ECG monitoring or clinical manifestations were observed in the remaining patients.

### Treatment

Five patients with incomplete communicating or noncommunicating cysts in the MRC group were recommended to take surgical treatment. Another 8 patients with complete communicating cysts were not recommended to take surgical intervention. In the surgical group, all 10 patients underwent surgery without consideration of this MRC classification. Table 2 shows the surgical information from the 2 groups.

### Follow-Up

All patients were continuously followed up. MRI was regularly checked, whether or not they underwent surgery. No patients presented with the symptoms of contrast agent residue, allergic reactions, or neurological impairment. All of the patients who received surgery did not develop complications such as intracranial infection or intracranial hemorrhage. We also evaluated cyst size. We calculated the percentage shrinkage of cysts pre- and postoperation. The dates from 2 groups were compared with statistical software. Although significant differences were not found between them, the percentage shrinkage in the MRC group was larger than in the surgery group. The results are shown in Tables 4 and 5.

**TABLE 4:**
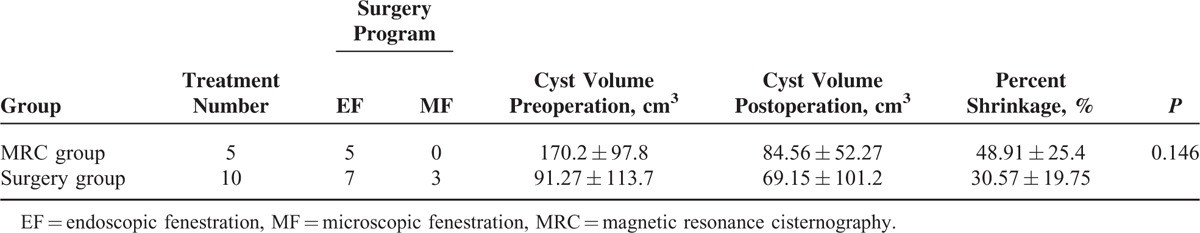
Treatment and Follow-Up Information of MRC and Surgery Group

**TABLE 5:**
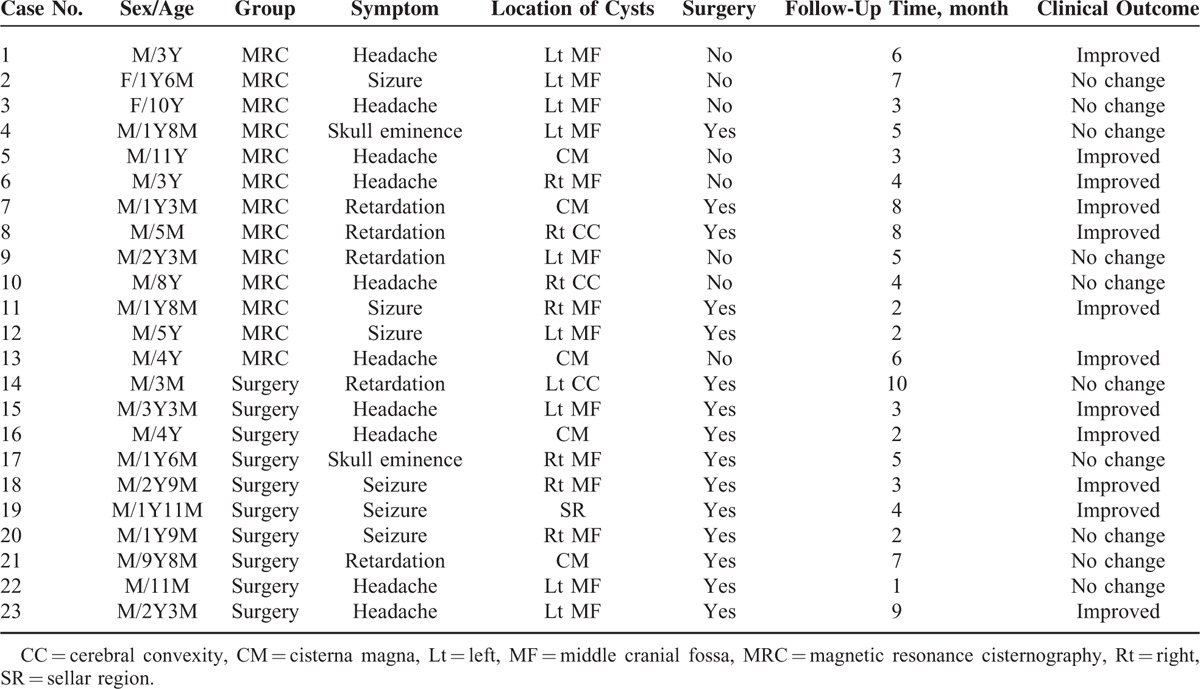
Clinical Characteristics With Follow-Up Result

## DISCUSSION

In the current study, 13 symptomatic ACs accepted MRC and were classified into 3 subgroups based on MRC results. We applied surgical treatment in the 3 subjects with incomplete communicating cysts and 2 subjects with noncommunicating cyst in the MRC group. In the 5 patients who underwent operations, the cysts had shrunk by the time of the follow-up investigation. By comparing the percentage shrinkage of the 5 patients and surgical group without MRC, there was no significant difference between them. However, we did not perform surgery in 8 patients in the MRC group. We concluded that the use of MRC with Gd-DTPA as a contrast agent is of significant clinical value for the diagnosis and treatment of children with intracranial ACs. The results of our study showed that the classification using dynamic MRC might have potential in decision of surgical treatment.

### Safety of the Gd-DTPA Contrast Agent for MRC

Gd-DTPA, a paramagnetic contrast agent, has been widely used in angiography.^13^ If Gd-DTPA is intrathecally injected, the relaxation time of T1 and T2 imaging of cerebrospinal fluid can be shortened. At a low concentration, Gd-DTPA mainly affects T1 images. At a high concentration, it mainly affects the T2 relaxation time.^14^ Intrathecal injection of Gd-DTPA during MRC was first performed on laboratory animals by Di Chiro in 1985.^15^ In subsequent years, researchers carried out a series of studies on other animals and on humans.^10,16^ These studies have suggested that the intrathecal injection of Gd-DTPA is a relatively safe procedure. At a diagnostic dose, it has no significant effect on the nervous tissue and does not cause significant side effects. In the published studies, very low concentrations (eg, 0.17 μmol/g of brain tissue) of Gd-DTPA are sufficient to clearly show the structure of the spinal cord and intracranial subarachnoid space.^11^ According to these published data from clinical studies, we controlled the dosage of Gd-DTPA from each patient, administering doses equivalent to 0.17 μmol of gadolinium element per gram of brain tissue.^11^ Until now, only limited information has been available concerning these procedures in children. To further avoid or reduce the side effects of drugs, we emphasized strict control of the injection velocity at 1 mL/min. The vital signs, rashes, convulsions, and other acute adverse reactions were closely monitored for 24 hours after successful cisternography. According to the observations, no patients had serious side effects or complications correlated with MRC. Slight transient headache was the only symptom, which was observed in only 1 patient. The symptom was relieved within 24 hours after bed rest and the oral administration of analgesics. During the follow-up after cisternography, the patients did not complain of any adverse reactions. Their parents did not find any adverse reactions, either. This is consistent with previous reports.^17–19^ In this regard, we have begun to continue the follow-up to investigate the long-term impact of Gd-DTPA on children.

### The Advantages of MRC Over Other Tests

In the past few decades, intrathecal iodide-enhanced CTC and radionuclide cisternography have been the most commonly used methods to evaluate the flow pathways of cerebrospinal fluid in the cistern or ventricles. However, a growing number of issues, such as ionizing radiation, the interference of skull artifacts, the limitations of axial plane imaging (multiplanar imaging is impossible), and allergies to the contrast agent, have been raised. Therefore, many centers tend to replace the former 2 methods with noninvasive special MRI sequences, such as 3D heavily T2-weighted sequences and phase-contrast (PC)-MRI of cerebrospinal fluid. Although 3D heavy T2-weighted sequences, represented by 3D constructive interference in steady state, can clearly show the surrounding anatomical structures of the cyst, they cannot provide information regarding cerebrospinal fluid flow. Compared with 3D heavily T2-weighted sequences, PC-MRI can provide important information regarding cerebrospinal fluid flow. When it is used for the diagnosis of cysts in the suprasellar cistern or the intraventricular area, where the change of cerebrospinal fluid flow is complex, the false-positive rate is greatly increased.^9,20^ Compared with the above-imaging methods, cisternography with Gd-DTPA as a contrast agent can make up any deficiencies. MRC can clearly show the anatomical structures on different planes and provide accurate information concerning the flow of cerebrospinal fluid.

### The Clinical Significance of Different MRC Results

During the examination of intrathecal Gd-DTPA MRC, ACs in different patients exhibited different enhancement features or commuting modes. We found that the contrast agent filled more slowly in incomplete communicating ACs. One possible explanation is that the entrance from the subarachnoid space to the cysts is small. Therefore, the contrast material enters the cysts slowly, and this slow process has also been reported in other study.^21^ Another possible explanation is that CSF can flow into the cyst quickly, but cannot flow out quickly because of one-way valve. We have not found that cysts with instant imaging and suspended regression of the contrast agent.^22^ We speculate that it can be the problem of the small number of subjects in this study. We also speculate that the incomplete communicating ACs with light terminal filling in the cysts are caused by the secretive function of the cyst wall because the cells of the cyst wall can move contrast materials from the outside into the cyst cavity. The secretive function of the ultrastructures and immunohistochemistry of the cyst lining cells in the cyst wall has been observed in many studies.^23–25^ We defined 3 types of ACs (complete, incomplete, and noncommunicating types) in our study according to the result of consecutive CTC. In our study, the definition of ACs has been used to the indication of surgery on the patients with ACs, and its value has been proven in the follow-up.

### The Influence of MRC on Surgical Decision-Making

Although the treatment of intracranial ACs is controversial, a growing number of studies have suggested that accurately understanding the communicating characteristics between the cysts and the subarachnoid space are very important in the choice of surgery.^4,7,26^ For example, surgery is necessary for patients with symptomatic ACs that do not communicate with subarachnoid space.^4,7^ If cisternography shows complete communication, which suggests favorable communication between the cysts and the subarachnoid space, regular follow-up is enough for the patients. If the cisternography shows incomplete communicating or noncommunicating ACs, as was the case in this study, patients would undergo surgery. In a patient with left temporal bone uplift, MRC showed delayed imaging, suggesting the slow communication between the cyst and the surrounding cerebrospinal fluid. Therefore, we made a surgical plan for that patient. On the basis of the results in this group, we believe that it is necessary to carry out rigorous and comprehensive analysis of the medical history, physical examination, and imaging tests, especially MRC, before performing surgery for AC cases. All 8 patients who did not undergo surgery exhibited symptoms. When also taking the results of MRC into account, we concluded that these patients’ symptoms were not necessarily caused by the cysts. Therefore, we chose to observe them further. During the follow-up, their symptoms were relieved. Repeated MRI did not show changes in the cysts. This also indicates that MRC is of significant value for the determination of surgical indications. The combination of clinical symptoms, signs, and MRC can reduce unnecessary surgery, decrease the risk caused by surgery, and alleviate the suffering of patients.^27,28^

We did not find a significant difference in the percentage shrinkage between these 2 groups. However, we found that the MRC group showed more shrinkage than the surgery group. The reason for this may be that some complete communicating ACs were included in the surgery group. Liquid inside and outside the cystic cavity can communicate freely in the patients with complete communicating ACs. There was no compression of the adjacent brain tissue by the cysts, and it is difficult for the brain tissue to return to a normal location after the surgery. In the MRC group, the patients with complete communicating ACs were excluded through the MRC before the surgery. We found the differences of volume between MRC and surgical groups. The sample size in this study was small because only children with intracranial ACs were recruited. The ACs were found in the middle cranial fossa, cisterna magna, cerebral convexity, sellar region, cerebellopontine angle, intraventricular region, and quadrigeminal region. In our data collecting process, the ACs were most commonly found in the middle cranial fossa and cisterna magna. Therefore, we chose these 2 types of ACs as our representative research focus. Furthermore, previous studies showed that the cysts shrank and the symptoms improved significantly after surgery.^4,7^ Future research with large numbers of patients and patients who received lumbar puncture or lateral ventricular puncture are required. However, this study might suggest that Gd-DPTA MRC is one safe and tolerable tool to make the decision of treatment for these patients with ACs.

## CONCLUSIONS

MRC should be applied to evaluate the status of ACs. The classification of ACs using dynamic MRC can benefit the surgical decision-making. For example, not all the symptomatic patients with complete communicating ACs were required to take surgical treatment.
